# Safe and Efficacious Near Superhydrophobic Hemostat for Reduced Blood Loss and Easy Detachment in Traumatic Wounds

**DOI:** 10.1021/acsami.3c12443

**Published:** 2024-01-19

**Authors:** Yibing Dong, Yaoxian Xu, Chengxing Lian, Krisna Prak, Hwa Liang Leo, Teresa D. Tetley, Vania Braga, Mike Emerson, Josefin Ahnström, Choon Hwai Yap

**Affiliations:** ‡ Department of Biomedical Engineering, National University of Singapore, Singapore 117583, Singapore; § Department of Bioengineering, Imperial College London, London SW7 2AZ, United Kingdom; ∥ National Heart and Lung Institute, Imperial College London, London SW3 6LY, United Kingdom; ⊥ Department of Immunology and Inflammation, Imperial College London, London W12 0NN, United Kingdom

**Keywords:** superhydrophobic, hemostasis, carbon nanofiber, blood coagulation pathway, toxicity, bandage, trauma

## Abstract

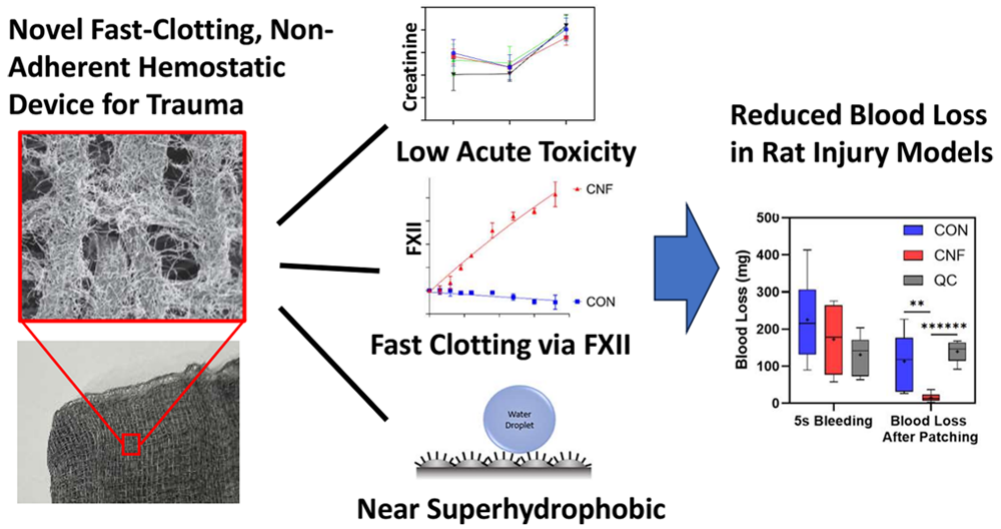

Hemorrhage is the leading cause of trauma death, and innovation in hemostatic technology is important. The strongly hydrophobic carbon nanofiber (CNF) coating has previously been shown to have excellent hemostatic properties. However, the understanding of how CNF coating guides the coagulation cascade and the biosafety of CNF as hemostatic agents has yet to be explored. Here, our thrombin generation assay investigation showed that CNF induced fast blood coagulation via factor (F) XII activation of the intrinsic pathway. We further performed studies of a rat vein injury and demonstrated that the CNF gauze enabled a substantial reduction of blood loss compared to both the plain gauze and kaolin-imbued gauze (QuikClot). Analysis of blood samples from the model revealed no acute toxicity from the CNF gauze, with no detectable CNF deposition in any organ, suggesting that the immobilization of CNF on our gauze prevented the infiltration of CNF into the bloodstream. Direct injection of CNF into the rat vein was also investigated and found not to elicit overt acute toxicity or affect animal survival or behavior. Finally, toxicity assays with primary keratinocytes revealed minimal toxicity responses to CNF. Our studies thus supported the safety and efficacy of the CNF hemostatic gauze, highlighting its potential as a promising approach in the field of hemostatic control.

## 1 Introduction

Trauma is the leading cause of death of individuals under the age of 45 in the U.S. according to the American Association for the Surgery of Trauma in the United States of America (U.S.A.).^1^ Trauma injuries encompass a wide range of incidents, including road accidents, industrial accidents, battlefield injuries, and intentional acts of violence, such as stabbing and shooting accidents and crimes, making them a prevalent global issue. Each year there are 1.3 million deaths from road accidents,^2^ 213 000 battlefield deaths,^3^ and 2.3 million workplace accident deaths worldwide.^4^

Among trauma-related injuries, acute hemorrhage is a leading cause of death.^5^ The ability to control bleeding immediately at the site of injury during the critical “platinum 5 min”, i.e., during the first 5 min post-injury, plays a crucial role in determining the mortality outcome and whether severe complications, such as amputations, occur.^6,7^ Disturbingly, 87% of battlefield deaths occur before arrival at a medical facility.^8^ Thus, it is imperative to develop more effective devices for on-site hemorrahage control. This is of particular importance when many trauma deaths are deemed potentially survivable and are related to hemorrhage. For example, 24.3% of battlefield mortalities were deemed potentially survivable, and these were predominantly (91%) caused by hemorrhage.^8^ An autopsy study of civilian pre-hospital trauma death in the U.S.A. reported that hemorrhage caused 34% of deaths, while combined hemorrhage and neurotrauma caused another 15% of deaths. Close to a third (29%) of these civilian trauma deaths were judged potentially survivable, of which 54% were primarily due to hemorrhage.^9^

Cotton gauzes have long been utilized as a topical hemostatic material owing to its safety, cost-effectiveness, and ease of application.^10^ For pre-hospital trauma application, which is the interval between the injury and when the victim arrives at the hospital, cotton gauzes are still the predominant device used today. However, its hydrophilic nature often results in the absorption of a significant volume of blood before the clotting is achieved and bleeding ceases, and after clotting, dressing removal often results in reopening of the wound and secondary bleeding. Many advanced gauzes have been developed in recent years to speed up clotting to enhance hemorrhage control, such as QuikClot Combat Gauze, Celox Rapid Gauze, and HemCon ChitoGauze. These devices rely on kaolin or chitosan impregnated into the gauze to accelerate clotting, but they nonetheless absorb substantial amounts of blood before clotting has occurred and have strong adhesion to the wound after clotting.^11−13^ Most recently, a zeolite hemostatic gauze has been developed and obtained U.S. Food and Drug Administration (FDA) approval. It has been shown to exhibit pro-coagulant effects for blood loss and hemostatic performance.^14−16^ Montmorillonite is another effective hemostat among natural phyllosilicates.^17^

In our previous work, we have reported a hydrophobic immobilized carbon nanofiber (CNF) coating that can serve as a novel and effective hemostatic device.^18,19^ It confers several advantages suitable for pre-hospital trauma victims. Its high repellence of water prevents loss of blood through absorption of blood by the gauze; the nanofibers cause fast clotting; and after clotting, the material has an extremely low adherence to the wound and could be easily detached without disturbing the newly sealed wound. This property is important because dressing change or removal often causes secondary wound tearing and bleeding, which can be dangerous to trauma victims because they are likely to have depleted clotting blood factors.^20^ In fact, adherence of dressing to wounds resulting in pain and trauma has recently been assessed to incur substantial wound management costs.^21^

However, the coagulation mechanism provided by CNF and the toxicity profile of a CNF-coated gauze were not fully understood, and efficacy in a severe injury was not studied. Therefore, in this study, we investigated the biosafety of CNF-coated gauze with cellular and animal studies, validated its efficacy in animal studies, and demonstrated that CNF induces clotting through coagulation factor (F) XII activation.

## 2 Materials and Methods

### 2.1 Materials

Vapor-grown CNF (719811; purity, 98%; diameter, 100 nm; and length, 20–200 μm), kaolin (K7375), and dichloromethane were purchased from Sigma-Aldrich. The 96-well plates (CytoOne plate flat bottom) were purchased from Starlabs and Corning. Human plasma (STA-Routine QC) was purchased from Stago. Non-woven gauzes were purchased from Melintex Pharma Co., Ltd. and Smith & Nephrew Pte Ltd. Polydimethylsiloxane (PDMS, Slygard 184) was purchased from Dow, and PDMS (MDX4-4210, Liveo) was purchased from DuPont.

### 2.2 Hydrophobic CNF Coating

To coat non-woven gauze, PDMS was dissolved at 2% in dicholoromethane and ultrasonicated with a probe ultrasonicator at an amplitude of 30% for 10 min. Non-woven gauzes were dipped in 2% PDMS in dicholoromethane solution for 5 min and baked at 100 °C for 30 min for PDMS coating. Following by that, CNFs and PDMS were individually dispersed in dichloromethane using ultrasonification (QSONICA Q500) at room temperature for 25 and 10 min, respectively. The two mixtures were combined and sonicated for an additional 5 min before being sonicated for another minute in the presence of a curing agent. The resulting composite dispersion was then spray-coated onto PDMS-coated gauze for animal study and baked at 80 °C for 1 h. To coat 96-well plates (CytoOne plate flat bottom, Starlabs, U.K.), CNFs were dispersed in 100% isopropanol with ultrasonication for 25 min and then added to wells using a multichannel pipet for 200 μL per well. The plate was incubated at room temperature in a fume hood with the lid open for 24 h. To remove any loosely attached CNFs, the coated plates were exposed to compressed air from a spray gun for 1 min.

### 2.3 Characterization of CNF/PDMS-Coated Gauze

#### 2.3.1 Field Emission Scanning Electron Microscopy (FESEM)

The surface morphology of CNF/PDMS-coated gauzes were examined by FESEM (JEOL JSM-6340F and 7600F, Tokyo, Japan). Each sample was placed on a scanning electron microscopy (SEM) stub with carbon tape and coated with gold by sputtering at a current of 20 mA for 30 s. The acceleration voltage was set to 5 kV.

#### 2.3.2 Water Contact Angle (WCA)

The hydrophobicity of various hydrophobic CNF gauzes was evaluated by measuring the contact angle using distilled (DI) water in a customized experimental setup. The static CA was determined following the sessile drop method by pipetting liquids onto flat substrates. A volume of 10 μL was employed for the experiments, and the images were captured by a digital camera. The images were analyzed by the drop shape analysis method using ImageJ.

#### 2.3.3 Static Immersion Test

Following the guidelines outlined in standard BS 34491, we conducted a slightly modified static immersion test to assess the water and blood absorption capabilities of various types of gauzes.^22^ Dry gauzes measuring 20 × 20 mm were carefully weighed using a 24-well plate. Using tweezers, each gauze was fully immersed in a beaker containing either deionized (DI) water or citrated porcine blood for a duration of 1 min. Subsequently, the wet gauzes were allowed to hang and air dry for 2 min before being reweighed. The percentage absorption of water and blood for each gauze, denoted as % Abs, was calculated using the following equation. The calculations were performed for three repetitions, and the average values were recorded (*n* = 3).
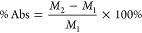
1From eq 1, *M*_1_ and *M*_2_ represent the mass of dry and wet gauzes, respectively. The smaller the water or blood absorption percentage, the more hydrophobic the gauzes.

#### 2.3.4 *In Vitro* Rat Blood Peeling Force

*In vitro* peeling force was performed using citrated rat blood to evaluate the force needed to detach the various kinds of gauzes via a modified procedure from Li et al.^11^ At a volume ratio of 10:1, citrated blood and 0.2 M CaCl_2_ were mixed to initiate coagulation. A total of 20 μL of blood was subsequently pipetted and sandwiched between two gauzes (15 × 15 mm) on a Petri dish. Following by that, a blood clot was allowed to form and solidify for 2 h at 37 °C. A thin cotton wire was glued onto the top gauze with epoxy and dried overnight. One side of the gauze was stuck onto a glass slide, while a force sensor was stuck to the back of glass slides to capture the force needed to peel and separate two gauzes attached together by a clot. The pulling motion was executed at a speed of 4.5 cm/min, and the force data were recorded using LabVIEW. The normalized gauze-peeling tension, NGPT, was computed with the following equation:
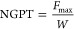
2where *F*_max_ is the maximum gauze-peeling force that is obtained at the largest clot width, *W*. The NGPT of each gauze was calculated using eq 2 and used for comparison between the different gauzes.

### 2.4 Hemostatic Mechanisms of CNF

#### 2.4.1 Human Plasma Clotting Assay

A total of 4 mg of CNF and kaolin powders were spread on double-sided tape, rolled along its length, and placed in a 96-well plate. Vials containing uncoated double-sided tape were used as the control (CON). PDMS films were rolled and used as a negative reference for clotting (PDMS). Coagulation was initiated by the addition of 20 mM CaCl_2_ to the platelet-poor human plasma (at a 1:9 volume ratio), resulting a final concentration of 2 mM CaCl_2_. The absorbance at 340 nm was measured continuously for an hour at room temperature and used as a quantification of fibrin deposition. The clotting time was defined as the time taken to reach half maximal clot formation from initiation of the reaction.

#### 2.4.2 Calibrated Automated Thrombogram (CAT) Assay

CAT assays were performed to determine the procoagulant potential of CNF in platelet-poor (PPP) and platelet-rich (PRP) plasma using a Fluoroscan Ascent FL plate reader (Thermo Scientific) and Thrombinoscope software (Synapse BF, Maastricht, Netherlands). For this, 96-well plates were coated by CNF, as described above. Both PRP and PPP were prepared using fresh human blood from healthy blood donors. Approval of the study was obtained from the Imperial College Research Ethics Committee (ICREC reference 20IC5940), and all the methods were performed in accordance with the relevant guidelines and regulations. All participants gave written informed consent to participate. The PPP from a total of 15 individuals was prepared, pooled, and stored in −80 °C as previously described.^23^ For PRP, whole blood was drawn from an individual donor into 10 mM sodium citrate and centrifuged at 150*g* for 10 min without any break. The PRP was stored at 37 °C until used and was assayed no longer than 2 h after blood sampling.

The CAT assays were performed in either PRP or PPP supplemented with 4 μM phospholipids (80 μL/well of both). The phospholipids (Avanti polar lipids) 1,2-dioleoyl-*sn*-glycero-3-phosphocholine (DOPC), 1,2-dioleoyl-*sn*-glycero-3-phosphoserine (DOPS), and 1,2-dioleoyl-*sn*-glycero-3-phosphoethanolamine (DOPE) were mixed in a molar ratio of 60:20:20 and extruded as previously described.^24^ Thrombin generation was initiated by the CNF coating after recalcification at 16.6 mM CaCl_2_ in a total volume of 120 μL/well. Thrombin generation (TG) was monitored using 0.42 mM Z-Glu-Gly-Arg-AMC (Bachem). To inhibit contact activation, corn trypsin inhibitor (CTI, Enzyme Research Laboratories) was added (65 μg/mL plasma). A 1 pM tissue factor (Dade Innovin), the initiator of the extrinsic pathway, was added to uncoated wells as a positive control. All concentrations given are final. The TG was monitored for 90–120 min. For analysis, lag time and time to peak were shown as 90 min (PPP) or 120 min (PRP) if no TG was recorded at the end of the assay.

#### 2.4.3 Chromogenic Substrate Assay for FXII Activity

FXII activation by CNF was investigated by incubating FXII in CNF-coated 96-well plates, followed by detection of FXIIa by a chromogenic substrate or western blotting. For this, CNF coating was performed by ultrasonicating 20 mg of CNF in isopropanol for 25 min, followed by pipetting 200 μL of the mixture into each well, and then allowing isopropanol to evaporate overnight. For FXII activation, FXII [200 nM (chromogenic assay) or 400 nM (western blot analysis), Enzyme Research Laoboratories] was diluted in 20 mM Tris at pH 7.5 and 150 mM NaCl (TBS) and was added to both CNF-coated and uncoated wells. FXII was incubated in the plate at 37 °C and removed at time points ranging from 0 to 180 min. After incubation, FXIIa was either quantified by a chromogenic substrate (on the basis of activity) or detected by western blot. For the chromogenic assay, 90 μL of each sample was mixed with 10 μL of chromogenic substrate H-d-Pro-Phe-Arg-pNA (0.5 mM, S-2302, Chromogenix) in an uncoated 96-well plate and immediately measured for absorbance at 405 nm at 22 °C for 20 min. The generated FXIIa was quantified against a standard curve generated by known amounts of FXIIa (Enzyme Research Laboratories). FXII activation was also analytically analyzed by western blotting, where 15 μL of reaction was added to each well and run under reducing conditions. FXII(a) was detected using a polyclonal antibody against FXII(a) (ab242123, Abcam).

### 2.5 Rat Vein Injury Model

In this study, the rats were subjected to vein injury with reference to a previously described procedure.^25^ Male NTac Sprague Dawley rats weighing 400–500 g and aged 3–4 months were used to establish a vein injury model. The rats were housed in the National University of Singapore (NUS) Animal Holding Facility (MD1) under controlled conditions with a 12 h light/dark cycle and provided *ad libitum* access to food and water. All surgeries were conducted in a separate room during the light phase (from 0700 to 1400 h) and performed under isoflurane anesthesia. The research protocol (R22-0392) was approved by the Institutional Animal Care and Use Committee (IACUC) of NUS, adhering to the guidelines provided by the National Advisory Committee for Laboratory Animal Research (NACLAR). The rats were anesthetized with isoflurane (3–5% induction dose and 2–3% maintenance dose) in 100% oxygen. The left thigh fur was removed using hair removal cream, then cleaned, and disinfected. A 1.5 cm incision was made to expose the vein. A 0.5 mm incision was made on the vein using a 25-gauge needle. After a brief 5 s bleeding period, preweighed gauze was applied to the wound to capture any blood escaping from the site. Subsequently, pristine gauze (CON group) or CNF-coated gauze (CNF group) was placed on the wound, covered with a transparent dressing film (Tegaderm, 3M, Saint Paul, MN, U.S.A.). To achieve hemostasis, a 100 g weight was used to compress the wound for 30 s, and then it was left undisturbed for an additional 3.5 min. The total amount of blood loss was assessed by visually evaluating and weighing the gauze collected from the wound site and the preweighed gauze. Following the procedure, the rats were placed on hothands for recovery, and 100% oxygen was administered via a nose cone.

### 2.6 Rat Artery Injury Model

Experiments were further conducted with the more severe rat femoral artery injury model. Sprague Dawley rats weighing 275–300 g and aged 8 weeks were used to establish an artery injury model. Upon arrival of animals, they are quarantined and adapted to the environment for 5 days, and the overall health of the animals is monitored every day. The research protocol (KR-IACUC-ST-2023-039) was approved by Guangdong Jinshi Medical Technology Services Co., Ltd. The rats were put in a glass cup, briefly anesthetize by an isoflurane-dipped gauze, and maintained anesthesia with a breathing anesthesia machine. The skin of the thigh of the rat was shaved from the groin area. Using a surgical blade, the tissue was bluntly dissected to expose the femoral artery. The glass separator was used to isolate the femoral artery, and a small incision was made using a needle (*d* = 0.45 mm) at a point where there were no branches in the femoral artery. The length of the incision was one-third of the diameter of the femoral artery, and there was free bleeding for 30 s. The bleeding was measured using preweighed gauze; then the gauze was removed; and a hemostatic material was applied to cover the incision. A weight of 100 g was placed on the material as the standard applied pressure. After every 2 min, the weight was removed and the material was lifted to observe whether hemostasis was completed successfully. Hemostasis was considered successful if no bleeding occurred for 2 min after removal of the material. The weight of the material and gauze was recorded before and after hemostasis to calculate the amount of bleeding during patching.

### 2.7 Biocompatibility Assays

#### 2.7.1 Cell Viability

Normal human epidermal keratinocytes (nHEK) were seeded into a 96-well plate (167008, Thermo Fisher) for 5 × 10^3^ cells per well on a monolayer of 3T3 fibroblasts (7 × 10^3^ cells/well), previously treated with mitomycin C (4 μg/mL, M4287, Sigma-Aldrich), and cultured in 100 μL of FAD standard medium [FAD medium (custom-made, Gibco) containg 1× GlutaMAX supplement (35050061, Gibco), 5 ng/mL insulin, 10 nM cholera toxin, 0.5 μg/mL hydrocortisone, 10 ng/mL epidermal growth factor, and 10% fetal calf serum (FCS)] in a humid chamber at 37 °C and 5% CO_2_. After 50–60% confluency was reached, cells were treated with CNF from 1000 to 1 μg/mL in FAD standard medium containing 1% FCS for 24 h. Untreated and 0.1% (v/v) Triton X-100-treated cells were used as 100 and 0% viability controls, respectively. After 24 h, the medium was then replaced with Dulbecco’s modified Eagle’s medium (DMEM) phenol free (31053-028, Gibco) containing 1% FCS, 1×X GlutaMAX supplement, and 10% Alamar Blue (DAL1100, Invitrogen) solution. Cells were continued to culture for 3 h before the fluorescence was read at 560 nm of excitation and 590 nm of emission in a SpectraMax iD3 fluorometer. The cell viability (%) was calculated as (test sample – blank)/(untreated control – blank) × 100%. Three independent replicates were carried out for each assay. GraphPad Prism was used for statistics and data analysis.

#### 2.7.2 Determination of Apoptosis and Necrosis

Normal human epidermal keratinocytes (NHEK) were cultured the same way as for the cell viability assay but in a CellCarrier-96 ultra microplate for imaging (6055300, PerkinElmer). Cells were untreated or treated with CNF at 200 and 20 μg/mL. Staurosporine (0.1 μM) and Triton X-100 (0.1%) were used as apoptosis and necrosis controls. At 30 min before the 24 h treatment, cells were washed once with 100 μL of Annexin V binding buffer [10 mM 4-(2-hydroxyethyl)-1-piperazineethanesulfonic acid (HEPES) at pH 7.4, 140 mM NaCl, and 2.5 mM CalCl_2_] and incubated with 100 μL of Annexin V binding buffer containing 1% (v/v) Annexin V–FITC, 1% (v/v) propidium iodide [Annexin V–FITC apoptosis detection kit (ab14085, Abcam)], and 1:10 000 (v/v) Hoechst 33342 (Invitrogen, H3570) for 30 min. Cells were then washed once with 100 μL of 1× phosphate-buffered saline (PBS) and fixed with 50 μL of 4% paraformaldehyde (PFA, J61899.AK, Thermo Scientific) for 10 min at room temperature. Cells were washed with 1× PBS and covered with 100 μL of 1× PBS. The plate was imaged at random fields of view at 40× using an Opera Phenix high-content screening system (PerkinElmer). Images were visualized using ImageJ.

#### 2.7.3 Genotoxicity

NHEK culture for the genotoxicity assay were carried out the same way as cell apoptosis and necrosis assay, except a Gamma H2A.X staining kit (ab242296, Abcam) was used. Cells were untreated or treated with CNF at 200 and 20 μg/mL for 24 h. DNA double-strand break (DSB) inducer (1:150) was used as a positive control and treated only for 1 h. After the treatment, cells were fixed with 50 μL of 4% PFA for 10 min at room temperature. Cells were permeabilized and then stained with 1:100 (v/v) Gamma H2A.X as the primary antibody, 1:100 (v/v) FITC conjugate as the secondary antibody, and finally with 1:10 000 (v/v) Hoechst 33342 according to the manufacturing protocol. Cells were washed with 1× PBS and covered with 100 μL of 1× PBS. The plate was imaged at random fields of view at 40× using an Opera Phenix high-content screening system (PerkinElmer). Images were visualized using ImageJ.

### 2.8 Animal Study Assays

#### 2.8.1 Complete Blood Count (CBC) and Blood Chemistry

The acute systemic toxicity of the vein injury model was evaluated in rats. The POS group underwent a similar surgical procedure without vein puncturing but received an intravenous injection of CNF (0.5 mg/kg) dispersed in 0.1% Tween 80 into the vein. Blood and serum samples were collected at 1, 12, and 24 h after wound compression. Tail vein sampling (1 and 12 h) and cardiac puncture sampling (24 h) were performed to obtain whole blood and serum. Whole blood was collected in ethylenediaminetetraacetic acid (EDTA) collection tubes (0.25/0.5 mL MiniCollect Tube, Greiner, Kremsmunster, Austria) and analyzed using a veterinary hematology analyzer (Sysmex XN-V, Sysmex America, Lincolnshire, IL, U.S.A.) to determine the complete blood count, including red blood cell indices and the white blood cell differential profile. Another tube of whole blood was collected into a serum separator tube (0.5/0.8 mL MiniCollect complete tube, Greiner) and centrifuged, and serum was separated for analysis using a blood chemical profiler (Cobas C111 analyzer, Roche, Basel, Switzerland). The levels of creatinine, blood urea nitrogen (BUN), albumin, aspartate transferase (AST), and alanine transferase (ALT) were measured to assess potential toxic effects.

#### 2.8.2 Hemolysis

The cardiac puncture sampling (24 h) was performed to obtain whole blood and serum in the rat vein injury model for all groups. The rat blood was collected in sodium citrate collection tubes (3.2%, BD Vacutainer, Becton Dickinson, New Jersey, U.S.A.). The amount of hemolysis in rat blood was evaluated by quantification of hemoglobin (Hb) in the blood serum with the hemoglobin assay kit (MAK115, Sigma). The Hb standard solution used in this study had Hb concentrations of 20, 40, 60, 80, 120, 160, and 200 mg/dL, diluted from an initial stock of 500 mg/dL calibrator. Rat blood was collected and spun down immediately after collection to separate blood cells and serum. Blood serum was collected for hemoglobin quantification. A 50 μL sample solution was added to 200 μL of reagent, and samples were measured at an absorbance wavelength of 400 nm.

#### 2.8.3 Organ Histology

Rats were euthanized with an isoflurane overdose, and vital organs (heart, lung, liver, spleen, and kidney) along with tissues from the wound/injection site were collected. Tissue samples were drop-fixed using 10% neutral buffered formalin (Sigma-Aldrich), dehydrated in an ascending series of ethanol, cleared with xylene, and then embedded in paraffin wax. Sections of 5 μm thickness were cut and placed onto glass slides. The slides were dewaxed in xylene and hydrated with a descending series of ethanol before being stained with hematoxylin and eosin (H&E), after which the slides were dehydrated through an ascending series of ethanol to xylene before being coverslipped. Sections were obtained and mounted on uncoated microscope glass slides (Snowcoat, Leica Biosystems, Germany). After deparaffinization and rehydration, slides were stained with H&E. Tissues were examined using a fluorescent microscope (Olympus BX 51, Japan) for morphological analysis.

## 3 Results

### 3.1 Hydrophobicity of CNF/PDMS-Coated Gauze

**Figure 1:**
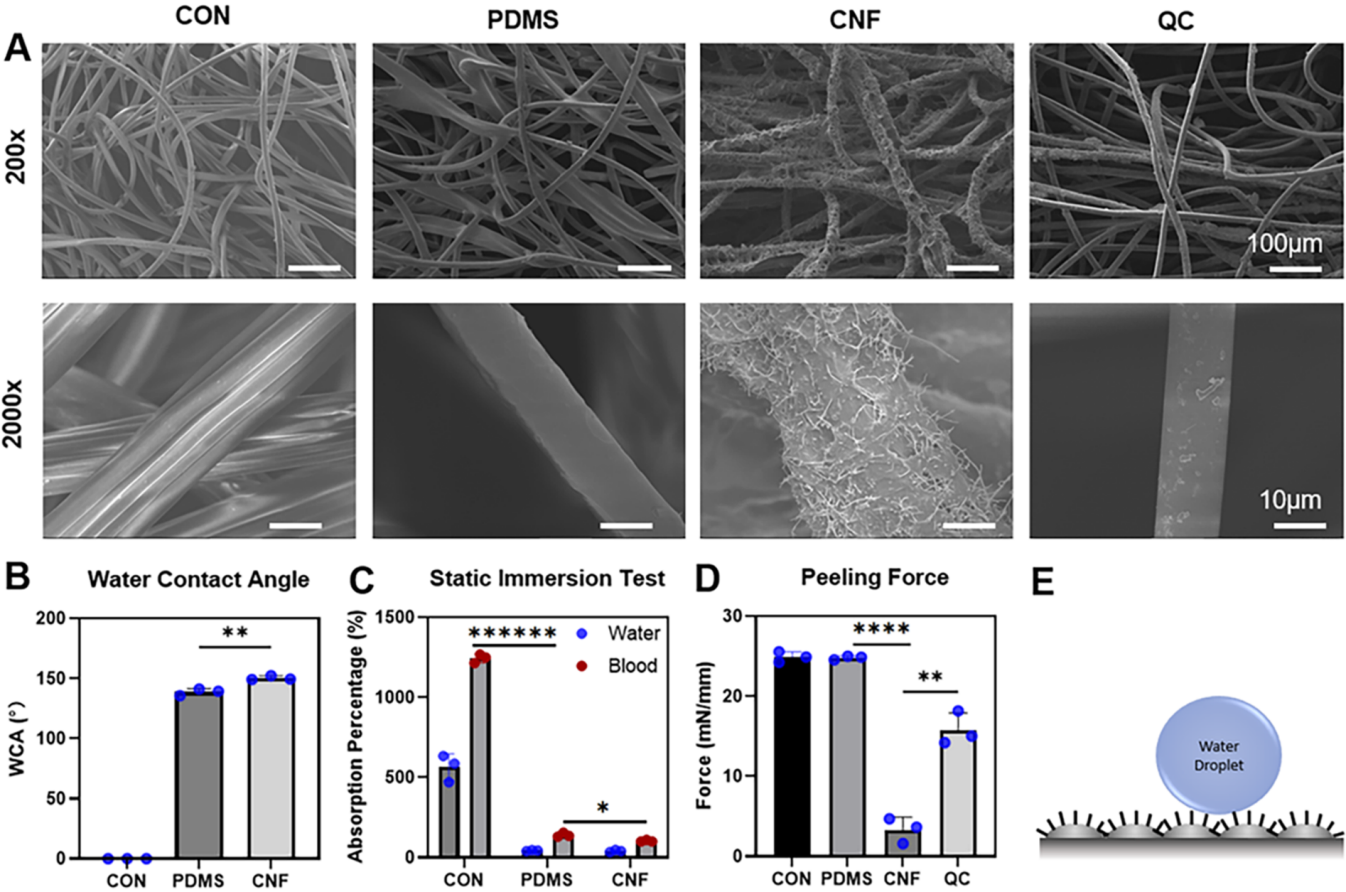
PDMS–CNF coating enables the hydrophobicity and low peeling force. Material characterization of PDMS-dipped and CNF/PDMS-coated gauze. (A) Material morphology by SEM of untreated gauze (CON), PDMS-dipped gauze (PDMS), PDMS-dipped and CNF/PDMS-spray-coated gauze (CNF), and QuikClot (QC), (B) contact angle measurements, and (C) static immersion test measuring water and blood absorption of the gauze, in terms of fluid absorption mass as a percentage of the dry gauze mass. (D) *In vitro* blood peeling force. (E) Illustration of CNF/PDMS-coated gauze in contact with water. Data are represented as the mean ± standard deviation. Results show that PDMS coating contributed to a hydrophobic surface of the gauze, while the lotus effect of CNF/PDMS coating enables the hydrophobicity and low peeling force of the gauze. The near superhydrophobic surface further reduced the peeling force of gauzes when in contact with blood. Data are represented as the mean ± standard deviation. Statistical significances are highlighted according to one-way analysis of variance (ANOVA), followed by the Bonferroni test (*n* = 3; ∗, *p* < 0.05; ∗∗, *p* < 0.005; ∗∗∗∗, *p* < 0.00005; and ∗∗∗∗∗∗, *p* < 0.0000005).

One essential advantage of using CNF to more traditionally used materials and gauzes is that it is hydrophobic, resulting in reduced blood loss and less risk of rupture of the newly sealed wound. As a first step in this study, the surface morphology of various gauzes was assessed by FESEM, and the results are presented. The surface morphology of various gauzes was shown in Figure 1. The cotton fiber bundles displayed a braided morphology as a base material (Figure 1A). After dip coating with PDMS, cotton fiber bundles appeared smooth and evenly covered with a hydrophobic PDMS layer, which provided a substantial increase to 138.4 ± 2.8° in the water contact angle (WCA) (Figure 1B) and imparted greater water repellency to the gauze. With a further spray coating of the CNF/PDMS mixture, CNF were immobilized onto the cotton fibers, creating nanoroughness on the cotton fibers (Figure 1E) that imparted hydrophobicity, high repellence to water, and a very high WCA (150.4 ± 1.8°, near superhydrophobic) (Figure 1B) and significantly reduced water and blood absorption during complete emersion in a small beaker of fluid at 1.5 cm of fluid depth (Figure 1C). When 20 μL of blood was coagulated between two of the tested surfaces, peeling forces were significantly reduced for CNF/PDMS-coated materials compared to untreated gauze, PDMS-dipped gauze, and QuikClot (Figure 1D). These results confirm that CNF/PDMS material thus has high water and blood repellency, confers easy detachment after clotting, and suggests that this is likely due to the lotus effect of surface nano- and microstructures of the gauze, where air plastrons repelled fluid and prevented adhesion (Figure 1E). The durability of hydrophobicity is assessed by the abrasion test (Figure S2 of the Supporting Information), where the near superhydrophobic surface was abraded with a plain cotton gauze under 10 kPa of weight. It could be observed that, despite a slight reduction in the contact angle initially, the overall contact angle only changed 4°, even when subjected to an abrasion distance of up to 10 m. This result demonstrates minimal shedding of CNF under mechanical abrasion.

### 3.2 Intrinsic Pathway (Factor XII Activation) Is the Main Mechanism for CNF Fast Clotting

The use of CNF has been shown to effectively induce clotting in one of our previous reports.^11^ However, for future clinical use, it is essential to understand the mechanisms behind the procoagulant/hemostatic effect of the CNFs. To this end, we performed pilot experiments using an *in vitro* clotting test with human plasma to determine the effects of CNF on the clotting time. Clotting of human plasma was initiated by the addition of CaCl_2_ and allowed for clotting for 30 min. Fibrin formation was measured at an absorbance of 340 nm. Half time was determined as the time at which the rate of plasma clotting was fastest. The results suggest that CNF reduced the clotting half time by nearly ^1^/_2_ compared to the blank well, control, and PDMS, indicating the procoagulant properties of CNF in the extracorporeal coagulation tests (Figure S1 of the Supporting Information).

With the results obtained that confirmed that CNF induces clotting of plasma, it was essential to determine the mechanisms behind this. We therefore next assessed whether CNF would induce thrombin generation in human plasma using calibrated automated thrombography. The advantages of using this method rather than the more clinically used measurements of clotting time is that they provide more information on the level and rate of thrombin generation. These assays are also more amenable for adjustments, enabling us to easily determine the molecular mechanisms involved in any procoagulant effect of our CNF. Thrombin is a critical enzyme at the center of the coagulation cascade and responsible for the formation of the fibrin meshwork that is required for the formation of a stable clot.

**Figure 2:**
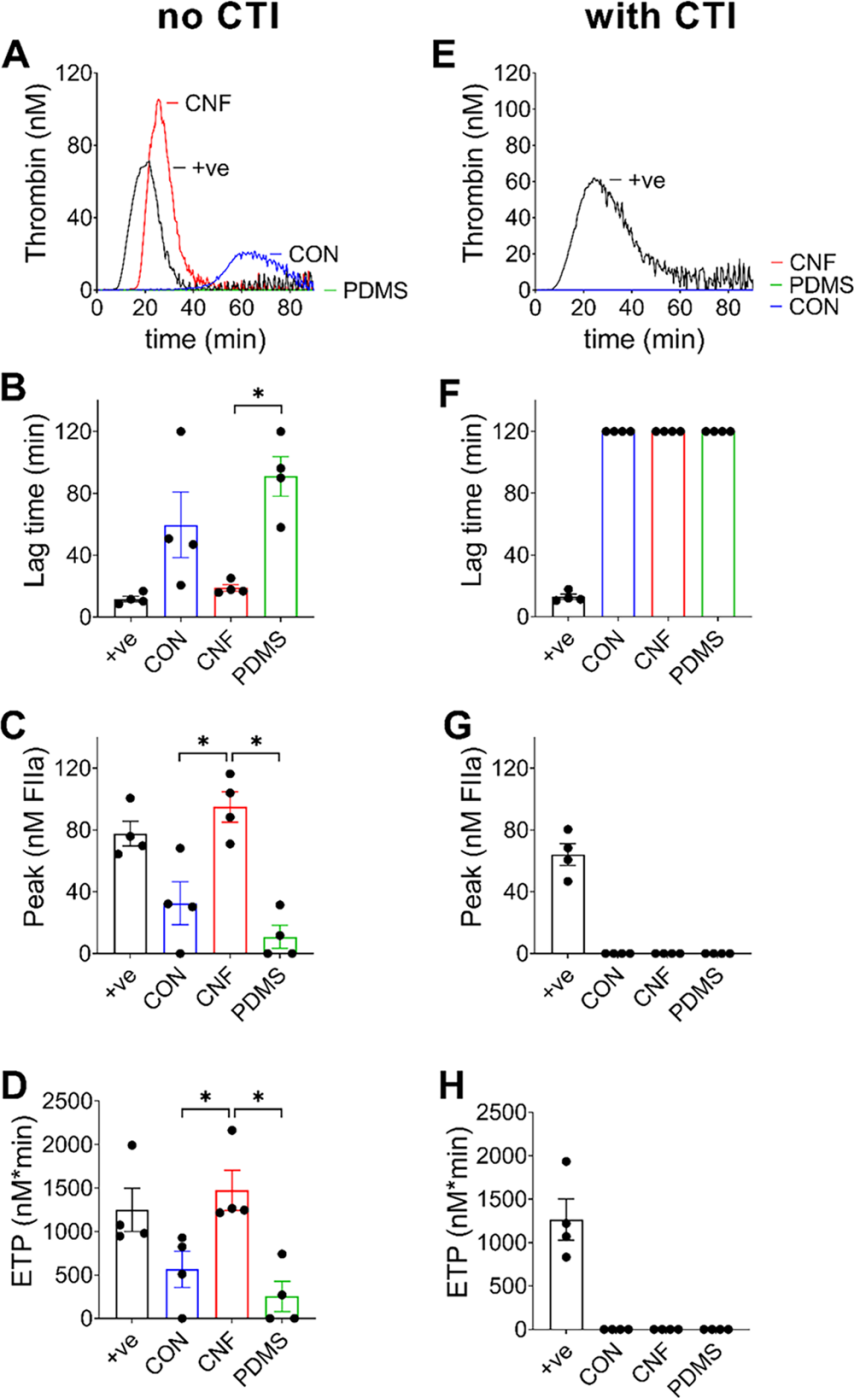
CNF induces thrombin generation in human PRP. Thrombin generation was assessed in PRP incubated in 96-well plates, where the wells were either uncoated (CON) or coated with CNF or PDMS in the (A–D) absence or (E–H) presence of CTI. (A and E) Representative thrombin generation curves are shown. The lag times (B and F) before thrombin generation and (C and G) peak thrombin generation and (D and H) endogenous thrombin potential (ETP, total amount of thrombin generated) are presented as the mean ± standard error of the mean of *n* = 4. +ve = positive control (1 pM TF). CON and PDMS were negative controls in this assay. Statistical significances, according to Mann–Whitney tests, between CNF and the negative controls (CON and PDMS) are highlighted (∗, *p* < 0.05).

For these experiments, recalcified PRP was added to 96-well plates coated with CNF and the thrombin generation was detected in real time using a fluorogenic thrombin substrate. Tissue factor (TF), the activator of the extrinsic pathway of coagulation, was added to PRP in uncoated wells as a positive control and, as expected, induced thrombin generation effectively (panels A–D of Figure 2). Uncoated wells and wells coated with PDMS, our negative controls, induced very little and severely delayed thrombin generation, as often seen in unstimulated PRP. In contrast, significant thrombin generation was observed in plasma added to wells coated with CNF, showing a procoagulant response to the material (panels A–D of Figure 2). In fact, we observed a ∼9-fold increase in the peak height of CNF compared to PDMS-coated wells (94.9 ± 19.7 versus 10.8 ± 14.9 nM; *p* = 0.029) and a ∼5-fold shortened lag time before thrombin generation (18.9 ± 4.2 versus 91.1 ± 25.6 min; *p* = 0.029), as presented in panels B and C of Figure 2. Physiologically, coagulation is often induced through the exposure of TF (lining the extravascular space) to blood upon vascular injury. TF induces coagulation through the initiation of the extrinsic pathway. However, in certain pathological situations, coagulation can also be induced by activation of FXII to FXIIa, the initiator of the intrinsic pathway, also called the contact pathway. This is the pathway targeted by various hemostatic products on the market, such as QC. To assess whether CNF induced thrombin generation through the intrinsic pathway, we supplemented the PRP with CTI, an effective inhibitor of FXIIa (panels E–H of Figure 2). As expected, our positive TF control still generated thrombin because coagulation here was induced by TF, which is not inhibited by CTI (panels E–H of Figure 2). In contrast, CTI efficiently inhibited the thrombin generation in the CNF-coated wells. The results showed that thrombin generation was induced through activation of the intrinsic pathway and most likely specifically through FXII activation (please compare panel A to panel E and panel C to panel G of Figure 2). Furthermore, these results also suggested that CNF was unable to induce coagulation through activation of a coagulation factor downstream of FXIIa and/or those in the extrinsic pathway.

**Figure 3:**
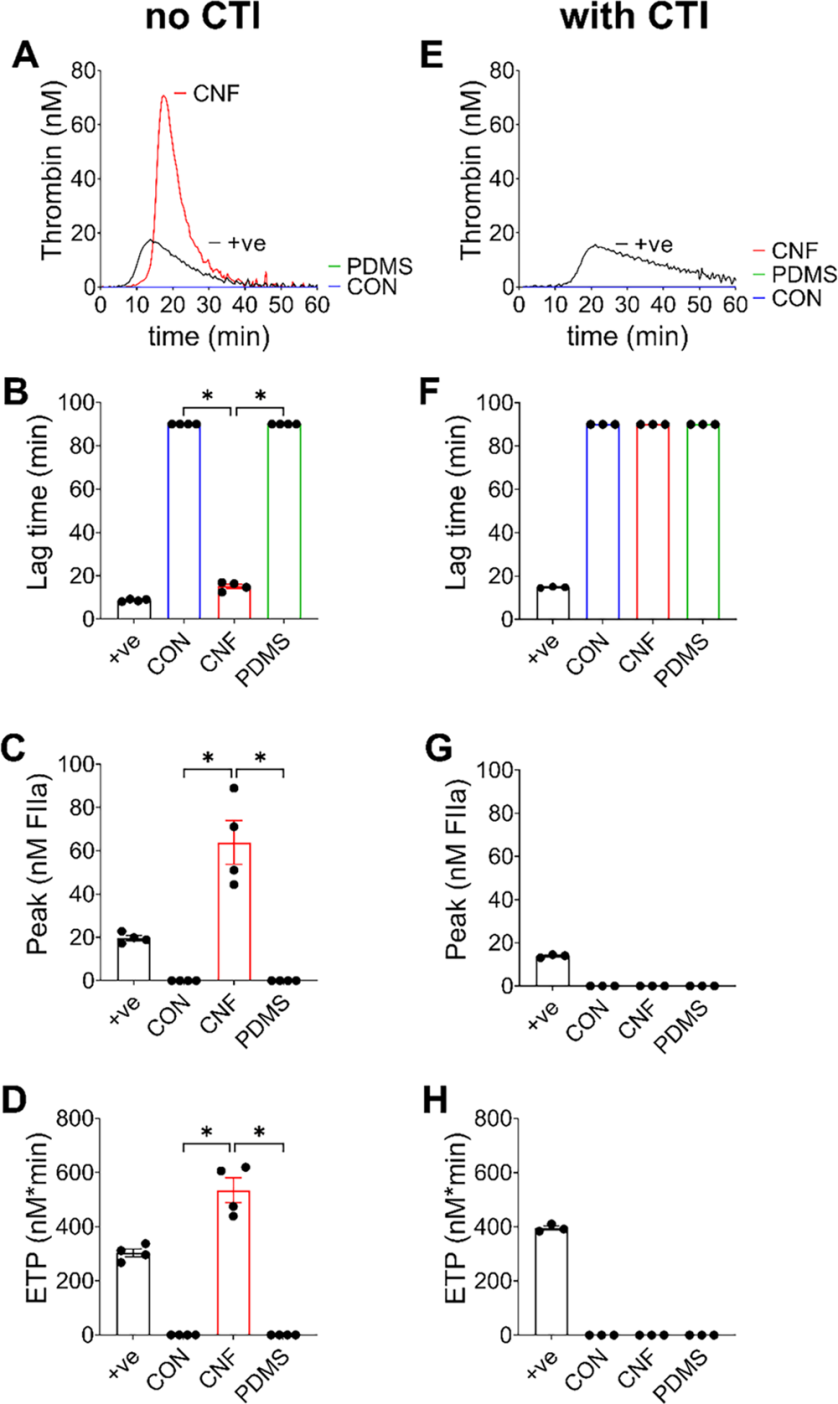
CNF induces thrombin generation in human PPP. Thrombin generation was assessed in PPP supplemented with 4 μM phospholipids and incubated in 96-well plates. The wells were either uncoated (CON) or coated with CNF or PDMS in the (A–D) absence or (E–H) presence of CTI. (A and E) Representative thrombin generation curves are shown. The lag times (B and F) before thrombin generation and (C and G) peak thrombin generation and (D and H) endogenous thrombin potential (ETP; total amount of thrombin generated) are presented as the mean ± standard error of the mean of *n* = 3–4. +ve = positive control (1 pM TF). CON and PDMS were negative controls in this assay. Statistical significances, according to Mann–Whitney tests, between CNF and the negative controls (CON and PDMS) are highlighted (∗, *p* < 0.05).

To assess whether the presence of platelets in PRP was essential for thrombin generation, the same assays were also repeated in PPP supplemented with synthetic phospholipid membranes that do not require activation (Figure 3). Importantly, no thrombin generation will occur during these conditions unless the coagulation cascade is initiated by activation of one of the coagulation factors. Also, in PPP, CNF induced thrombin generation (peak, ±20.2 nM; lag time, 15.1 ± 2.0 min), whereas no thrombin generation was observed in PPP added to wells that were either uncoated or coated with PDMS over 90 min (panels A–D of Figure 3). Similar to the results in PRP, the CNF-induced thrombin generation in PPP was blocked by the addition of CTI (panels E–H of Figure 3), once again showing that the thrombin generation was due to activation of the intrinsic pathway. Furthermore, because these results were performed in the absence of platelets, they show that platelets are not required for activation of coagulation by CNF, suggesting that CNF directly activates FXII specifically.

**Figure 4:**
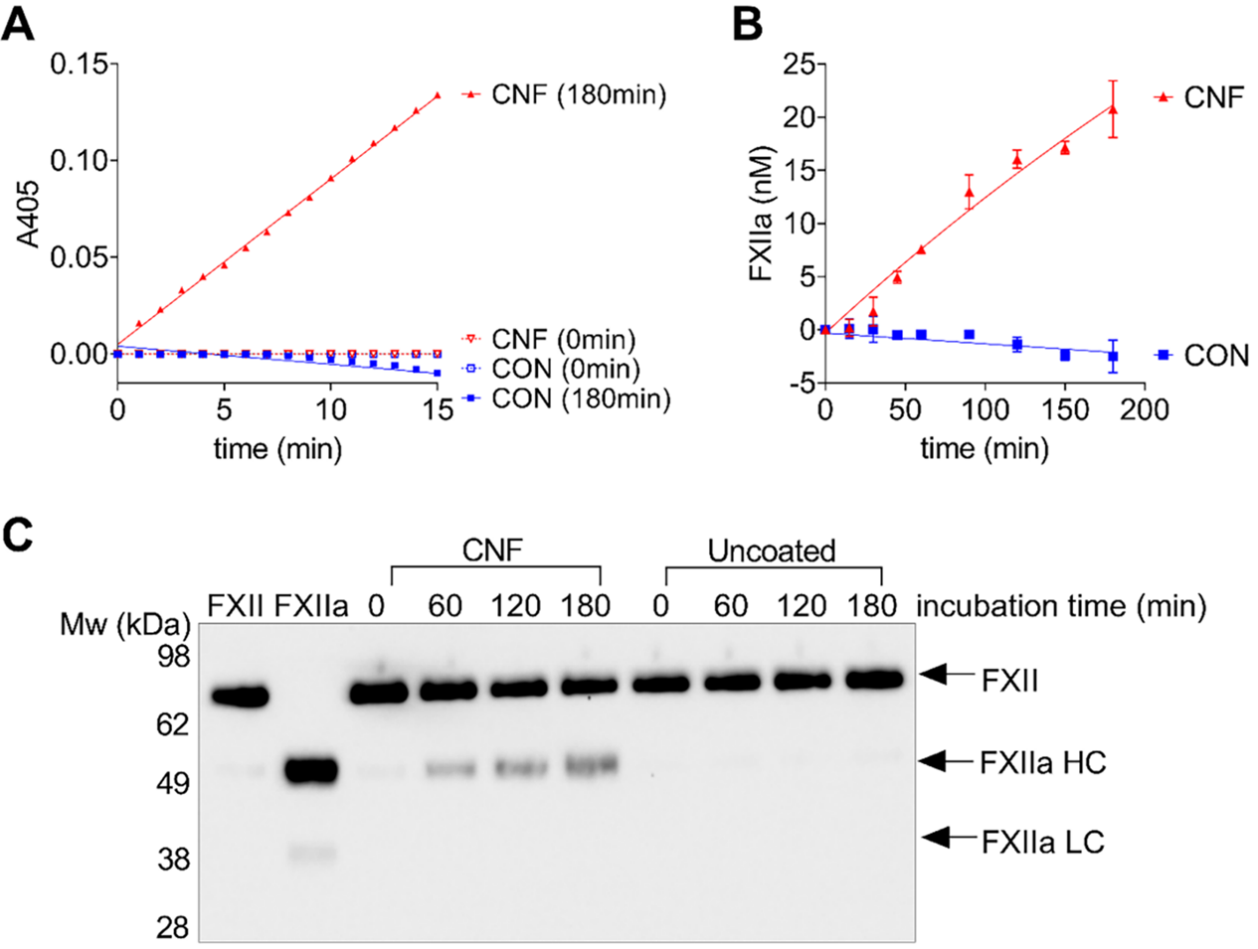
CNF activates FXII. FXII was incubated in 96-well plates with wells either uncoated (CON) or coated with CNF for 0–180 min. (A) Following incubation of FXII (200 nM), FXIIa activity was detected using a chromogenic substrate (0.5 M) at 405 nm. (B) FXIIa activity generated over time was quantified in comparison to a standard curve of known amounts of FXIIa. The results are presented as the mean ± standard deviation (SD) of *n* = 3. (C) Western blotting of the FXII samples (400 nM) collected at different time points confirmed activation of FXII. The arrows indicate FXIIa heavy chain (HC) and light chain (LC).

To further test our hypothesis that CNF induces thrombin generation in plasma by activating FXII, we performed pure-component FXII activation assays, where FXII activation was assessed using either a chromogenic peptide substrate or western blotting. Here, FXII was incubated in 96-well plates, where the wells were either uncoated or coated with CNF up to 180 min. When the samples were analyzed for FXIIa activity using a chromogenic substrate, absorbance at 405 nm was detected in samples incubated with CNF, whereas no absorbance was detected in the FXII sample incubated in uncoated wells, showing that CNF induced FXII activation (Figure 4A). The level of FXII activation was time-dependent, with ∼20 nM FXIIa being detected after 180 min of incubation (Figure 4B). FXII activation was confirmed by western blotting, where bands corresponding to the heavy and light chains of FXIIa were detected in the samples incubated with CNF only (Figure 4C). Together, these results show that CNF has a procoagulant effect through the activation of FXII to FXIIa, resulting in initiation of coagulation through the intrinsic pathway.

### 3.3 CNF/PDMS-Coated Gauze Reduces Blood Loss in a Rat Vein Injury Model

**Figure 5:**
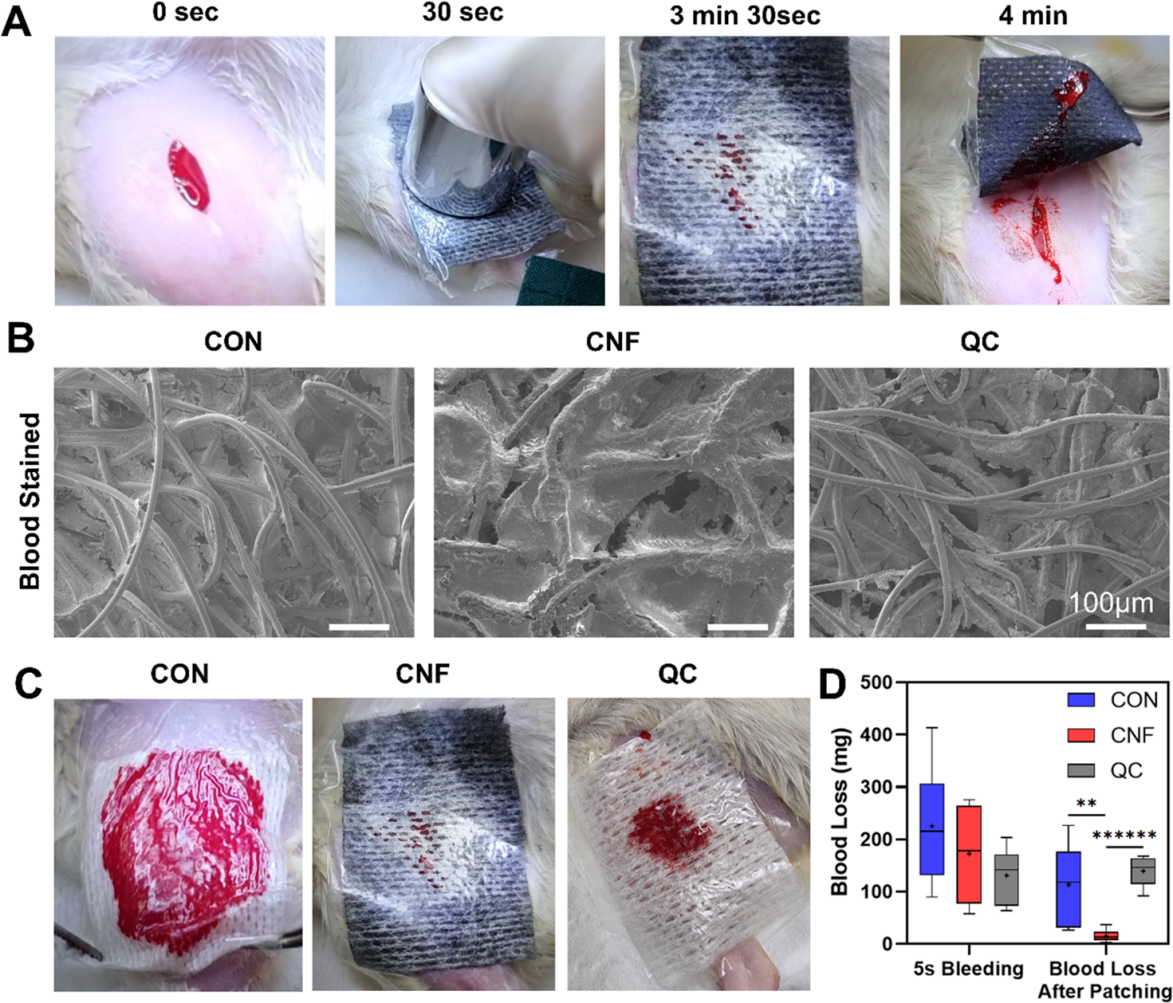
CNF-coated gauze reduced blood loss by over 80%in a rat vein injury model. (A) Experimental procedure of rat vein injury, with a 0.5 mm incision created in the rat vein, covered by gauzes with plastic backing, compressed by a 100 g weight for 30 s, left undisturbed for 3.5 min, and peeled off. Normal gauze and commercial product QuikClot were used as controls. (B) Blood stain on normal gauze (CON), CNF/PDMS-coated gauze (CNF), and QuikClot (QC) after peeling off. (C) At 3.5 min, the blood-soaked gauzes. (D) Box plots of blood loss in rat vein injury. Each data point represents the blood loss of one sample for each rat. Lower and upper fences are 25th and 75th percentiles, and the median cross is in between. Bars represent 10th and 90th percentiles. Statistical significances are highlighted according to one-way ANOVA, followed by the Bonferroni test (*n* = 6; ∗∗, *p* < 0.005; and ∗∗∗∗∗∗, *p* < 0.0000005). Results showed that the blood loss was significantly reduced in the CNF/PDMS-coated gauze group compared to uncoated gauze and QuikClot. A video of the procedure is given in Video S1 of the Supporting Information.

To verify the efficacy of near superhydrophobic CNF/PDMS gauze in hemostasis, studies were conducted in a rat model of saphenous vein injury, and the amount of blood loss with compression was assessed, comparing our CNF/PDMS gauze to the normal gauze control as well as the advanced QuikClot gauze. After the injury, 5 s of free bleeding was allowed, and the amount of blood loss by each of the three groups was not significantly different from each other, although substantial variability was encountered (Figure 5D). After another 30 s of gauze compression followed by 3.5 min of undisturbed clotting (Figure 5A), where the gauze was left on the wound without compression, bleeding stopped for all three groups.

When the patches were peeled off, the control cotton gauze was often thoroughly soaked with blood, while the QuikClot gauze had smaller blood stains, which is likely due to the QuikClot gauze being thicker (Figure 5C). For the CNF/PDMS-coated gauze, gauze fibers were not soaked with blood, but blood gathered in the pores and gaps between fibers, with the area of blood stain smaller than that of the QuikClot gauze. Weighing of gauzes before and after patching revealed that blood loss with the CNF/PDMS gauzes was significantly smaller than those on QuikClot and normal gauze groups (Figure 5C), being 89% less than the QuikClot group and 88% less than the normal gauze group. This demonstrated that the CNF/PDMS gauze had promising efficacy in blood loss reduction.

SEM images of the peeled gauzes (Figure 5B) showed that blood clots were well-mixed with the substrate fiber bundles for cotton and QuikClot groups, where blood clots were well beneath the surface and plain fiber bundles could be seen above the clots. For the CNF/PDMS gauze, however, clots were observed to cover fiber bundles, which could not be clearly observed, suggesting that the near superhydrophobic fiber bundles provided some resistance to blood penetration. Some blood clots, however, could be observed to penetrate in between fiber bundles.

### 3.4 CNF/PDMS-Coated Gauze Reduces Blood Loss in a Rat Artery Injury Model

**Figure 6:**
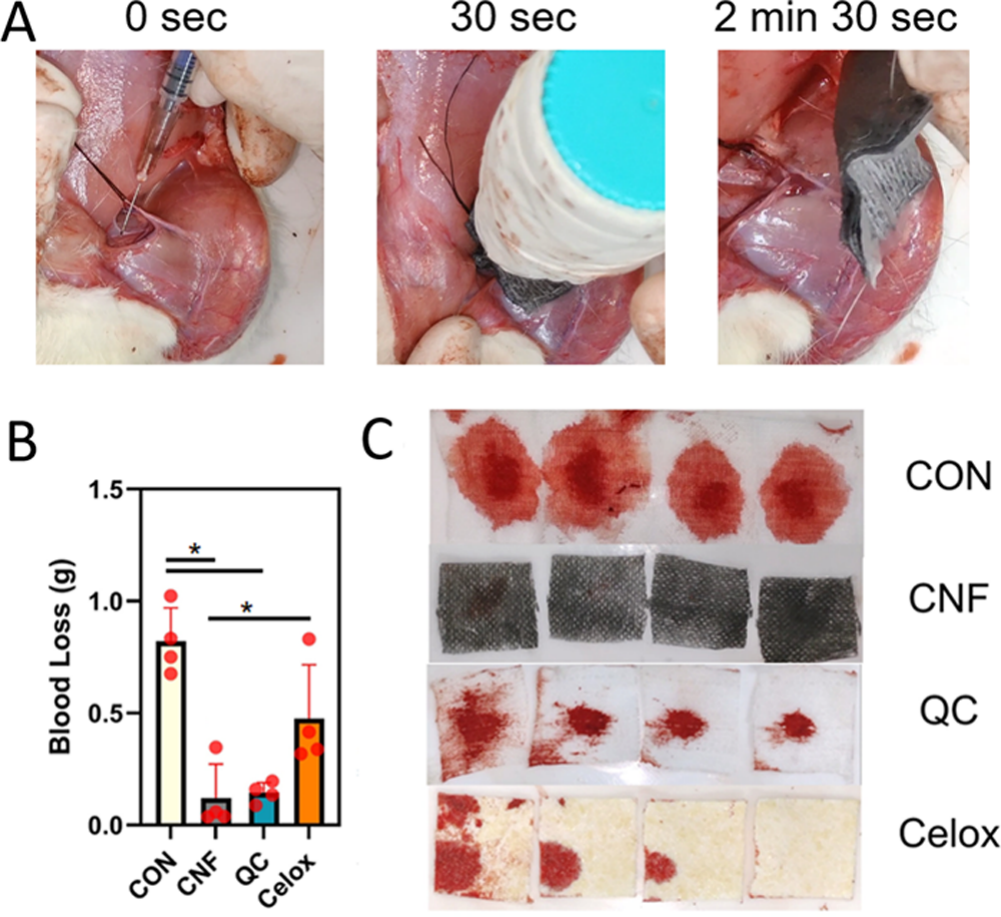
CNF-coated gauze reduced blood loss in a rat artery injury model. (A) Experimental procedure of rat artery injury, where a needle was used to injure the artery and gauzes were used to patch the wound with compression via a 100 g weight until hemostasis. All gauzes were four-layered. The gauze was then peeled off and weighed to measure blood loss. (B) CNF/PDMS gauze reduced blood loss significantly by 85% compared to the cotton control (CON) and 75% compared to Celox. It reduced blood loss by 17% compared to QC but without statistical significance. (C) Representative patch of each type after peeling off, with the four layers laid out side by side. Statistical significances are highlighted according to one-way ANOVA, followed by the Bonferroni test (*n* = 4; ∗, *p* < 0.05). Results showed that the blood loss was significantly reduced in the CNF/PDMS-coated gauze group compared to uncoated gauze and QuikClot.

A more severe bleeding model was conducted to compare CNF/PDMS-coated gauzes to the cotton control, QC, and Celox. The experimental procedure is shown in Figure 6A. An incision was made on the femoral artery, following by which the wound was allowed to freely bleed for 30 s. A standard weight was applied at the time of 30 s, and hemostasis was checked every 2 min until the bleeding stops. Results showed that the CNF/PDMS gauze can still reduce blood losses from cotton, QC, and Celox groups (Figure 6B), but the reduction is lower compared to the less severe rat vein injury model. Nonetheless, it can achieve 85% reduction from cotton gauze and 75% reduction from Celox, which were both statistically significant, and 17% reduction from QC, which was not statistically significant. This demonstrates good efficacy in a severe wound. In comparison to the other types of gauze, there is only one small blood stain on the gauze layers of CNF (Figure 6C).

### 3.5 Low Toxicity of the CNF Hemostatic Gauze

#### 3.5.1 Primary Keratinocyte Cell Toxicity Test

**Figure 7:**
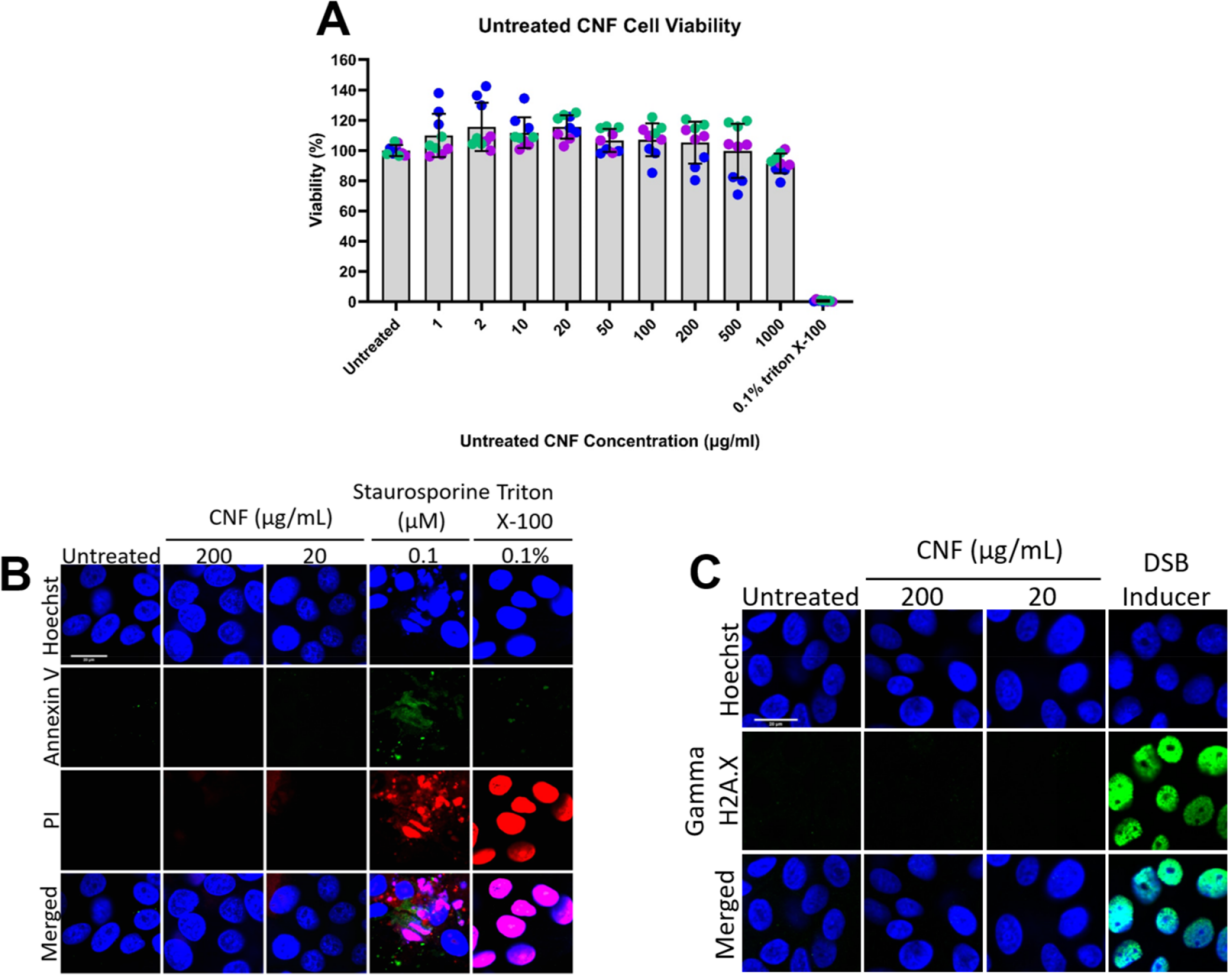
Assessment of CNF toxicity against nHEK. (A) Keratinocyte cell viability assay. Cells were incubated with different concentrations of CNF in media for 24 h, and the viability was determined using Alamar blue solution. Untreated cells were calculated as 100% viable. The viability of cells reduced to 0% when treated with 0.1% Triton X-100 for 24 h. (B) Apoptosis and necrosis assay, with confocal imaging of nHEK treated with 0.1 μM staurosporine (control for apoptosis), 0.1% Triton X-100 (control for necrosis), and CNFs for 24 h. Cells were live-stained with Annexin V (Annexin V–FITC, green) for the apoptosis marker and propidium iodide (PI, red nucleus) for the necrosis marker. (C) Genotoxicity assay. Confocal imaging of nHEK treated with a DNA double-strand break (DSB) inducer (control for DNA DSB) for 1 h and CNFs for 24 h. Cells were stained with γH2A.X/FITC (phosphor S139, green), with the nucleus in blue. Scale bar = 20 μm. The assays were performed in three replicates.

As mentioned above, for CNF to be therapeutically and clinically used as a hemostatic device, it is essential to confirm a lack of cell toxicity. To assess whether CNF could be toxic to human primary cells, the fibers were tested using normal human epidermal keratinocytes (nHEK). A 24 h period of testing is selected as a conservative duration for pre-hospital trauma hemostatic devices; they are expected to make skin contact within hours before patients reach a hospital. Primary human keratinocytes were used as a result of their sensitivity to injury and harmful stimuli, which have been shown to effectively indicate a significant response to low levels of toxic materials.^26−30^ Results in Figure 7A showed that, after 24 h incubation, there was no significant difference in nHEK viability between CNF-treated samples (1–1000 μg/mL) and the untreated sample, while treatment with 0.1% Triton X-100 reduced viability of the cells to 0%. Two concentrations of CNF at 20 or 200 μg/mL and 24 h of incubation with cells were selected for testing of apoptosis, necrosis, and genotoxicity. Annexin V stain for the apoptosis marker (Figure 7B) showed negative results for untreated and CNF-treated cells and a positive result for 0.1 μM staurosphorine, a positive control. In contrast to CNF, staurosphorine induced nucleus fragmentation and enhanced exposure to phosphatidylserine residues/Annexin V–FITC that indicated cell apoptosis. Figure 7B showed that, unlike 0.1% Triton X-100, CNF did not induce necrosis to nHEK, as indicated by propidium iodide staining. Genotoxicity analysis using phosphorylated gamma H2A.X as a marker of DNA double-strand break (Figure 7C) showed negative results when incubating the cells with CNF for 24 h. As a positive control, incubation of cells with a double-strand break inducer for 1 h showed that a striking amount of phosphorylated gamma H2A.X was induced. In summary, CNF is safe for human skin in the tested range of concentrations. The test was assessed for 24 h, which was a conservative duration for pre-hospital trauma hemostatic devices; however, patients are typically expected to reach the hospital within a few hours.

#### 3.5.2 Acute Systemic Toxicity in the Animal Model

After confirmation that CNF showed very little cell toxicity in cell-based assays, acute systemic toxicity was also tested *in vivo*. For this, we used a rat model, where the blood was drawn from the animals at 1, 12, and 24 h after the application of the gauze on the rat vein injury. An extensive analysis of blood samples was performed to detect acute toxicity of the CNF/PDMS gauze compared to the plain control gauze and the positive control, where 0.5 μg/kg CNF of body weight was injected into the vein.

**Figure 8:**
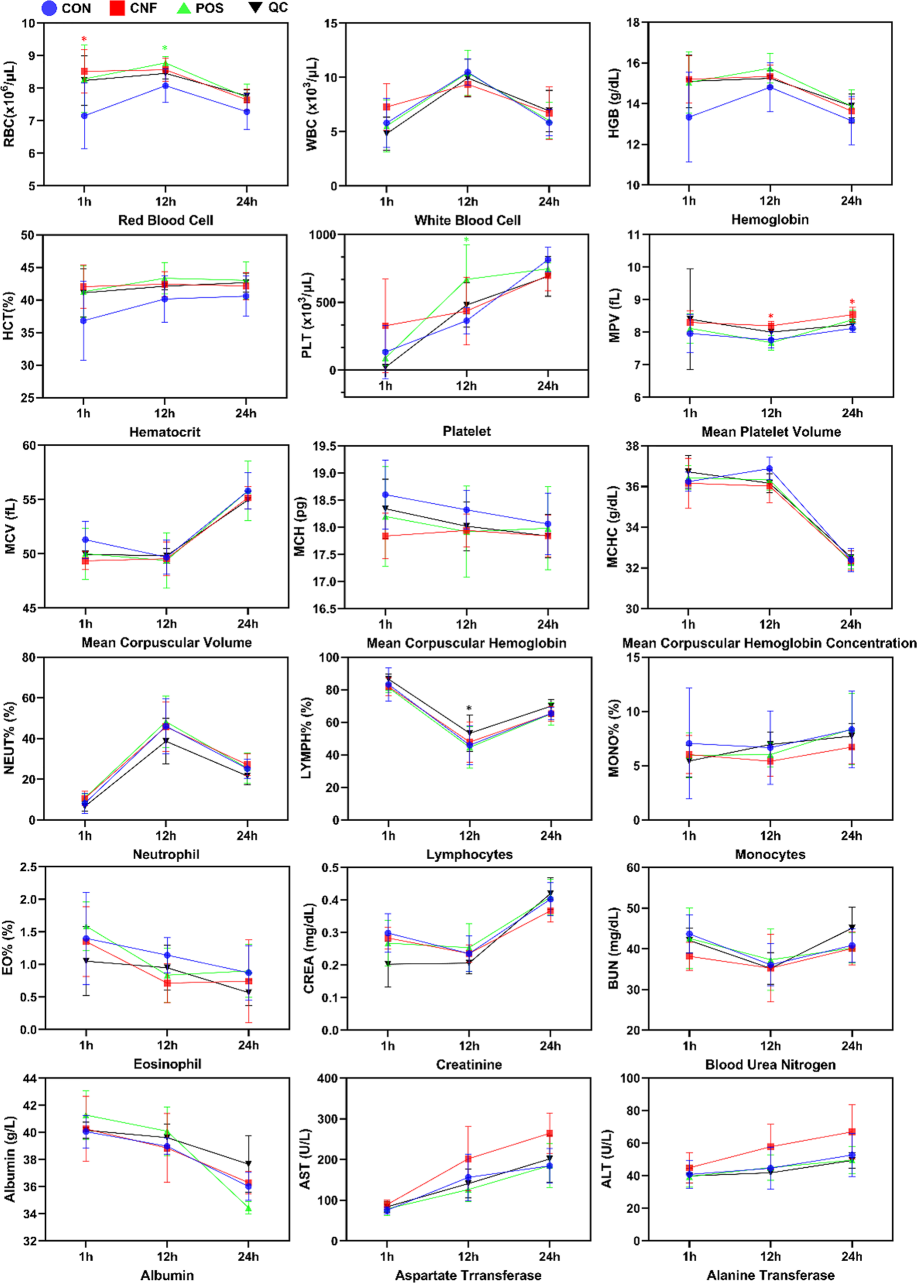
Hemocompatibility of CNF in the animal model of venous injury. Blood samples were collected from the tail vein at 1 and 12 h and via cardiac puncture at 24 h for assessment of hemolysis. Blood samples of animals from the CON (blue), CNF (red), POS (green), and QC (black) groups were further quantified for red blood cell indices, white blood cell differential profile, and blood chemistry. Data are represented as the mean ± SD, with *n* = 4 for CON at 1 h and CNF at 1 h for creatinine, blood urea nitrogen, albumin, aspartate transferase, and alanine transferase and *n* = 5 for all other groups (∗, *p* < 0.05 in comparison to the CON group).

Results are shown in Figure 8. Generally, for all groups, there was a mild decrease in red blood cells at 24 h, a transient increase in the white blood cell count and neutrophil at 12 h that was restored at 24 h to the level at 1 h, and a transient decrease in lymphocytes at 12 h that was similarly restored at 24 h. There was a gradual decrease in eosinophils, but no significant change to monocytes over the three time points. There were no significant changes to hematocrit and mean corpuscular hemoglobin across all time points, but corpuscular volume increased at 24 h. In terms of organ damage indicators, there were increases in creatinine (kidney function) and aspartate transferase (liver function) at 24 h, generally no significant changes to blood urea nitrogen (kidney function), and mild increases in alanine transferase (liver function) across all three time points and generally decreased albumin levels (liver and kidney function) at 24 h. These results suggested that the kidney and liver were under some stress at the 24 h time point, which could be attributed by the injury model, potential blood loss, and stress associated with the experiment.

**Figure 9:**
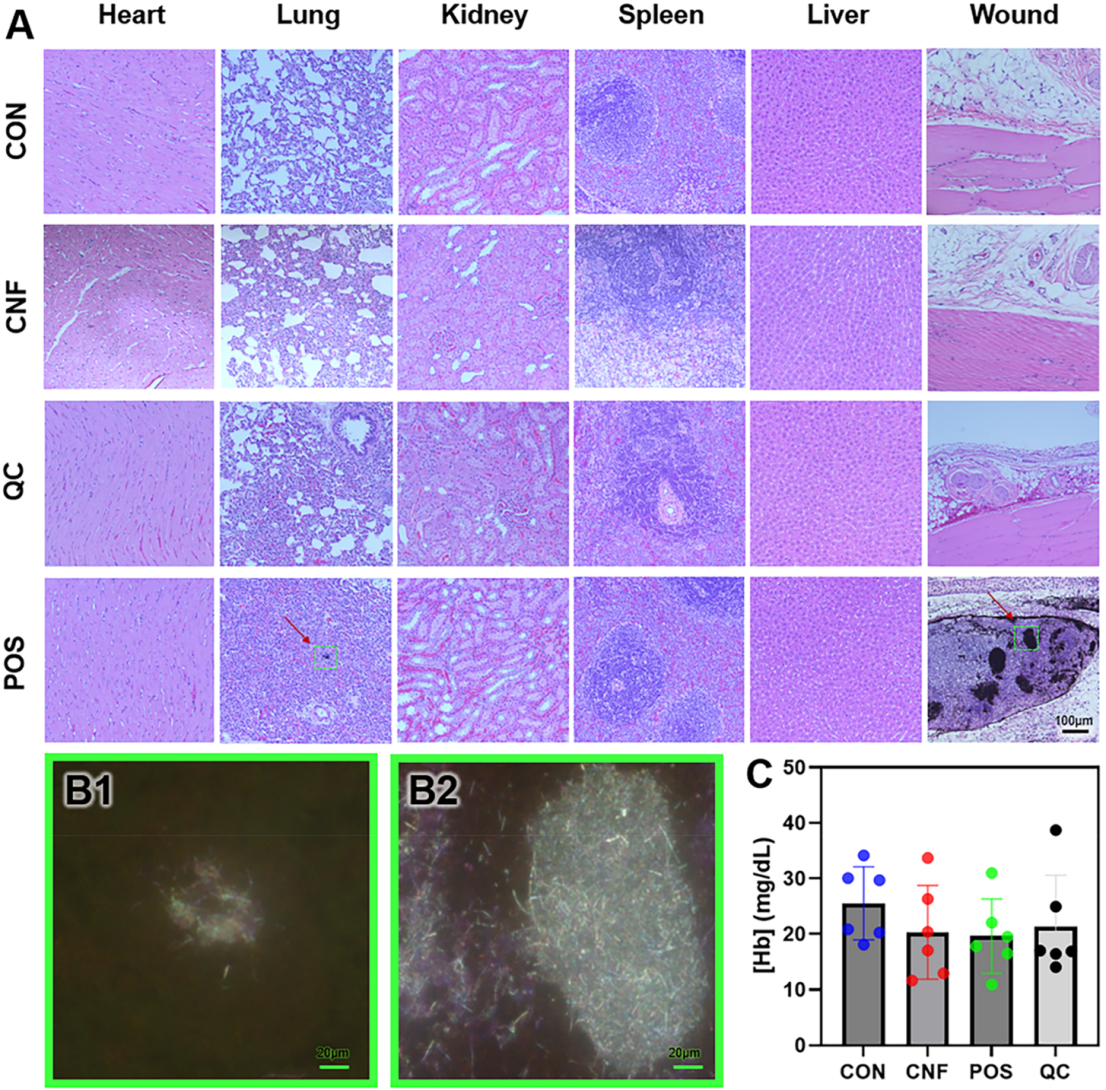
Acute systemic toxicity and hemolysis. (A) Bright-field images of H&E histological sections of major organ tissues at 24 h post-treatment, for the uncoated cotton gauze (CON), carbon nanofiber-coated gauze (CNF), QuikClot gauze (QC), and CNF injection group (POS). (B1 and B2) Dark-field images at the green box locations indicated in the H&E images of the POS group. CNF aggregates were detected in the (B1) lung and (B2) wound sites in the POS group. (C) Amount of hemoglobin detected in the blood serum, extracted 24 h after application of gauze and intravenous injection, demonstrating that no additional hemolysis was in the various experimental groups compared to cotton controls after the treatment. No significant difference was observed across groups according to one-way ANOVA, followed by the Bonferroni test (*n* = 6).

However, in comparison of the different experimental groups, no significant difference of the CNF/PDMS or QuikClot group from the normal gauze control group could be observed across all of the data presented (Figure 8), except for the red blood cell count at 1 h, where the cotton control group had a lower red blood cell count, which might be due to excessive blood loss. Further, the whole blood hemoglobin level of the cotton control group was lower than all other groups at 1 and 12 h, but differences were not significant. This could be related to the lower red blood cell count in the control group, and values in other groups did not exceed the expected normal range of 37 g/dL,^31^ suggesting no abnormality. Further investigation of the serum hemoglobin level at 24 h showed that it was not significantly different between the groups (Figure 9C), suggesting that no additional hemolysis occurred for the various experimental groups compared to cotton controls. These results suggested that the toxicity profiles of all of the gauzes investigated were not different from that of the normal gauze.

Interestingly, even the CNF injection group did not elicit overt toxicity, because blood analysis results demonstrated similar levels of cell count, whole blood hemoglobin level, organ function biomarkers (Figure 8), and serum hemoglobin level (Figure 9C). This corroborated previous results from Sachar et al., where single wall carbon nanotubes were observed not to cause hemolysis after prolonged incubation with red blood cells.^32^ Further, all rats in the injection group survived until euthanasia and did not exhibit noticeable behavioral changes, suggesting that CNF did not cause excessive acute toxicity.

As shown in Figure 9A, our H&E staining demonstrated no observable signs of acute organ damage in any of the groups compared to stains from non-treated control animals from the literature.^33,34^ Small aggregates were observed for the CNF injection group, such as in the lung and at the site of injection, appearing as black patches on H&E images, which were confirmed to be CNF via dark-field microscopy^35^ (Figure 9B), because they showed signals with fiber-like morphology co-localized with the black patches from H&E images (dark-field images were taken at the green boxed locations indicated on H&E images).^36^ At the wound site of the injection group, the presence of CNF appeared to trigger neutrophil infiltration. In the CNF/PDMS gauze groups, however, CNF was not found in the wound site histology or organ histology, suggesting that the immobilization of CNF on the gauze was effective in preventing CNF from infiltrating the bloodstream and depositing in organs.

## 4 Discussion

In this study, we provide evidence that the CNF-coated gauze has high efficacy as a trauma wound care device, with a good biosafety profile. We show that the fast-clotting mechanism of CNF involved the activation of FXII through the intrinsic pathway and provide cellular and animal data demonstrating low toxicity.

Our investigation supports the feasibility of a new class of hemostatic medical device for pre-hospital trauma, where hydrophobic devices with clot-inducing agents immobilized to the surface could be used together with compression to achieve fast clotting while minimizing soaking blood losses and, at the same time, enable extreme ease of detachment after clot maturation to minimize secondary bleeding during dressing change. The nanofibrous surface was previously shown to resist bacteria attachment, enhancing sterility of the device,^11^ and previous work has also shown that it is possible to add hydrophilic hemostatic compounds on top of the immobilized nanofiber layer to achieve even faster clotting without sacrificing ease of detachment.^19^ While CNF is the only nanofiber demonstrated to support such devices currently, it is likely that a number of other nanofibers can achieve similar performances.

We conducted experiments in two different injury models, a less severe vein injury model and a more severe arterial injury model, and found good efficacy for the CNF/PDMS gauze in both experiments. Collectively, our results suggested that having hydrophobicity properties on top of fast-clotting properties can be useful in both types on injuries, because they reduced blood losses in both experiments. Hydrophobicity could minimize blood losses through soaking of the gauze, while fast clotting enabled fast stabilization of the wound to reduce blood loss. However, our data suggested that the relative contribution of these two effects may vary with the wound severity. In the less severe vein injury model, the fast-clotting property alone (that of QuikClot) did not significant reduce blood loss, but the combination of fast-clotting and hydrophobic properties (that of CNF/PDMS gauze) drastically reduced blood loss. This showed that, in less severe injuries, gauze soaking was a dominant mechanism of blood loss that can be prevented with hydrophobicity. In the more severe arterial injury model, however, fast-clotting properties alone (that of QuikClot) provided significant reduction of blood loss from control gauzes, and the additional property of being hydrophobic (that of CNF/PMDS gauze) provided smaller further reduction in blood loss. Fast-clotting properties are thus relatively more important to reducing blood loss in more severe wounds, although hydrophobicity is still useful.

In the current study, we used a different gauze design from our previous implementation,^18,19^ converting the cotton gauze into a hydrophobic substrate via a dilute PDMS dip-coating treatment before immobilizing CNF on its surface. This modification resulted in reduced blood uptake and *in vivo* blood loss and was a good way to reduce blood penetration through the hydrophobic coating. During the application of the gauze, such as during handling or when compressed against the wound, it can easily be stretched at some locations, which can cause exposure of the underlying substrate to blood at these locations. Without the PDMS substrate coating, the substrate would absorb much blood as a result of this contact, rendering the nanofiber coating ineffective. Preventing the breach of the hydrophobic layers also ensured that the easy detachment property could be maintained. Furthermore, as a result of the low concentration of PDMS in the dip-coating solution, the cotton substrate remained very flexible after the coating and the final fiber-coated device did not feel different from the original substrate during handling. Substrate flexibility is important for some wound applications, such as packing deep wounds, and is an improvement upon current advanced bandages, such as QuikClot, which are often stiff and hard.

PDMS-coated cotton gauze is only one of several possible hydrophobic textile designs to achieve the above effects. There are likely several polymeric synthetic textiles that will also work well here, including polymers commonly used in biomedical applications, such as polyethylene (PE), polytetrafluorethylene (PTFE), and polyurethan (PU).^37^ PDMS, however, is of low cost, has good flexibility, ease of manufacture, good biocompatibility, and good gas permeability and liquid impermeability,^38^ and is thus an attractive option. It allows for aeration and help to maintain a healthy environment for the wound area, is hydrophobic and suitable for our hydrophobic gauze design, and can withstand long durations of storage without degrading.

Our results showed that CNF can initiate coagulation via activation of coagulation FXII. This is the same mechanism as that utilized by kaolin for accelerated clotting, which is used in QuikClot combat gauzes.^39^ FXII is generally seen as being autoactivated to foreign surfaces.^40^ In our case for CNF, it is possible that surface charges on CNF cause the autoactivation. This fast-clotting property is likely contributing to the substantial blood reduction compared to normal gauze in our rat experiment. The Fast-clotting property, together with the hydrophobicity of our gauze combined with proper compression that prevented soaking blood loss, could reduce blood loss by more than 80% from QuikClot in the vein injury model and by 17% in the artery injury model. These findings highlight the potential of CNF-coated gauze as a promising solution for trauma injury applications.

Importantly, our study examined the biosafety of CNF at the cellular and systemic levels and demonstrated low acute toxicity profiles. We observed that keratinocytes exhibited a cell viability over 70% after 24 h of incubation, and thus, they can be considered to be non-cytotoxic according to ISO 10993-5 standards.^41^ It is further important to note that the CNF gauze will most likely be applied for a much shorter duration than 24 h for the pre-hospital trauma application, because the dressing will be removed when the patients reach the hospital, and that only a small fraction of fibers is likely to be left behind in contact with keratinocyte as a result of fiber immobilization. Our assays for apoptosis, necrosis, and genotoxicity were also negative. The *in vivo* studies further showed that there was no observable acute systemic toxicity following the application of the CNF gauze, because blood analysis and histology did not reveal differences compared to the cotton control gauze group and no infiltration of CNF into the bloodstream was detected. Although the animal studies showed changes in blood count and organ function markers over time, they were observed for all groups and were overall within the normal ranges. The increase in white blood cells is likely a response to the injury of the vein,^42^ caused by acute inflammation, while the temporary increase in creatinine is likely to be due to isoflurane intake, which reduces urine output and creatinine clearance.^43^

## 5 Conclusion

This work therefore provides evidence that the CNF/PDMS-coated gauze has the potential to be a safe and efficacious trauma hemostatic device. In the future, further assessment for chronic toxicity responses to the CNF/PDMS-coated gauze and assessments of *in vivo* injury models of other injury types, such as internal organ wounding, may be helpful to understand the safety profile of the gauze. It is also essential to test the hemostatic devices in larger animal models to mimic the real-life scenario more closely. Our current work provides a detailed mechanistic understanding of CNF-initiated hemostasis and provides a new option for the field of hemostatic material for blood reduction in substantial bleeding.

## Supporting Information Available

Recording of the whole hemostatic process of CNF gauze for the vein injury model (Video S1) (MP4)
Human plasma clotting assay (Figure S1) and coating durability test (Figure S2) (PDF)
