# Supplementary material for: Safe and Efficacious Near Superhydrophobic Hemostat for Reduced Blood Loss and Easy Detachment in Traumatic Wounds
Source: ACS Appl Mater Interfaces. 2024 Jan 19;16(4):4307–20. doi: 10.1021/acsami.3c12443 (PMC10835652; doi:10.1021/acsami.3c12443)
Supplement: Supplementary file 2 — am3c12443_si_002.pdf [file am3c12443_si_002.pdf]

Supporting Information

A Safe and Efficacious Near Superhydrophobic Hemostat for Reduced Blood Loss and Easy Detachment in Traumatic Wounds

Yibing Dong[1]†, Yaoxian Xu[2]†, Chengxing Lian[2]†, Krisna Prak[3], Hwa Liang Leo[1], Teresa Tetley[3], Vania Braga[3], Mike Emerson[3], Josefin Ahnström[4]*, Choon Hwai Yap[2]*

[1] Department of Biomedical Engineering, National University of Singapore, Singapore
[2] Department of Bioengineering, Imperial College London, UK
[3] National Heart and Lung Institute, Imperial College London, UK
[4] Department of Immunology and Inflammation, Imperial College London, UK
† Authors have equal contribution.
* Corresponding Authors

Addresses for Correspondences:

Choon Hwai Yap, Ph.D.,
Depart of Bioengineering,
Imperial College London White City Campus,
Rm 923, Sir Michael Uren Hub,
86 Wood Lane, London W12 0BZ
Email: c.yap@imperial.ac.uk

Josefin Ahnström
Department of Immunology and Inflammation,
Imperial College London Hammersmith Campus,
Rm 5S5, Commonwealth Building,
Du Cane Road, London W12 0NN
Email: j.ahnstrom@imperial.ac.uk

1. Human plasma clotting assay

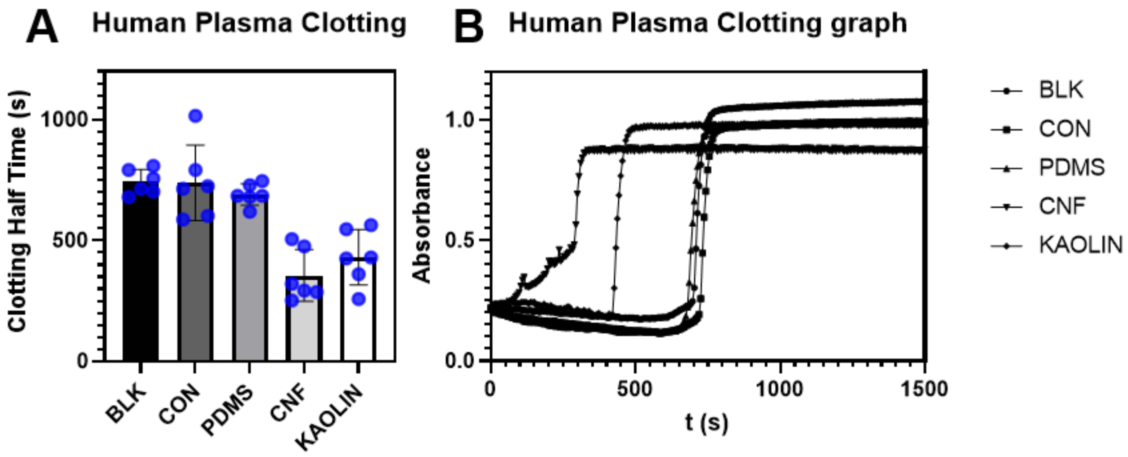

Figure S1. Human plasma clotting assay*. (A)** *Clotting half-time was assessed at the point of steepest slop of fibrin formation curve for empty well (BLK), double-sided tape (CON), PDMS-coated double-sided tape (PDMS), CNF-coated double-sided tape (CNF), Kaolin-coated double-sided tape (KAOLIN).* ***(B) *Fibrin formation curve at the absorbance of 340nm. The results indicate PDMS does not initiate clotting whereas CNF has a comparable speed in clotting initiation compared to Kaolin, offering pro-coagulant effect.*

2. Coating Durability Test

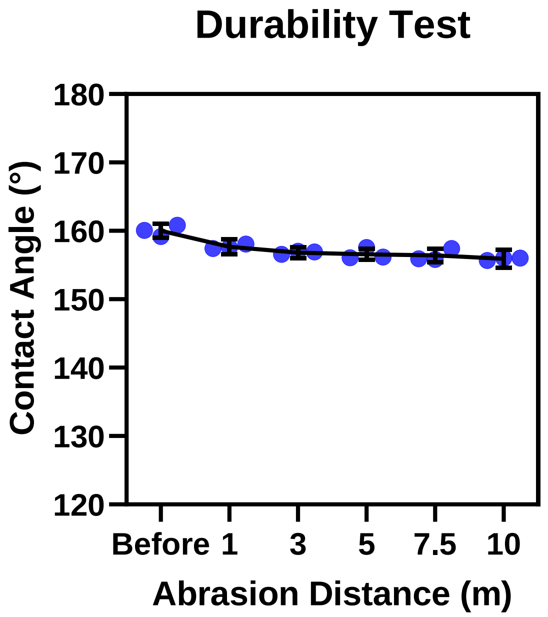

Figure S2. *An abrasion durability test was conducted to examine the mechanical durability of CNF-coated non-woven gauze samples. An untreated gauze served as the abrasion interface, with the CNF-coated gauze facing the abrasion interface. A pressure of 10 kPa was applied to the samples while they moved back and forth at a constant speed of 1 cm/s, covering a total distance of 10 m. Contact angle measurements were taken before the test and after each 1, 3, 5, 7.5, and 10 m of abrasion (n=3). After the test, no significant changes in contact angles were observed in the samples, and all of them maintained their superhydrophobicity with contact angles exceeding 150°.*
